# General practitioners’ relational familiarity with home-dwelling patients in the palliative phase: Basis – or bias – for individualised care

**DOI:** 10.1177/26323524261426398

**Published:** 2026-02-28

**Authors:** Katrine Staats, Sandra Jahr Svendsen, Veronica Lockertsen

**Affiliations:** 1 Faculty of Health Science, Department of Nursing and Health Promotion, OsloMet – Oslo Metropolitan University, Kjeller, Norway; 2 Lillestrøm Municipality, Lillestrøm, Norway

**Keywords:** relational familiarity, person-centred care, palliative care, home-dwelling, general practitioner, secondary analysis

## Abstract

**Background:**

An ageing population has increased the need for home-based palliative care. General practitioners (GPs) are central to continuity of care, yet time pressure and organisational constraints can challenge person-centred practice grounded in relational familiarity with patients and families.

**Objectives:**

This study aims to explore how GPs navigate the relational familiarity in home-based palliative care and whether it functions as a basis or a bias for individualised care.

**Design and methods:**

A secondary hermeneutic analysis was conducted, utilising data from two qualitative studies involving 21 GPs. These datasets focused on dignity-preserving care and shared decision making in palliative contexts. The hermeneutic circle was applied in an iterative process to interpret the in-depth interviews and focus group discussions.

**Study Design:**

A qualitative, explorative, and descriptive design grounded in hermeneutic methodology.

**Results:**

Two overarching themes emerged: (1) Relational familiarity: A cornerstone of complex dimensions, where familiarity enabled trust, continuity and tailored care; however, it also created overfamiliarity that narrows perspective, blurs boundaries and burdens GPs. (2) Variability in engagement and follow-up, which reflected differences in individual GPs’ interests, systemic constraints and the involvement of other healthcare professionals. While relational familiarity facilitated person-centred care, it was unevenly distributed, creating disparities in the quality of care.

**Conclusion:**

Relational familiarity is key to person-centred palliative care, fostering trust and continuity. However, it can blur boundaries, create emotional strain and contribute to inequities compounded by systemic challenges. Interdisciplinary collaboration and flexible care models are needed to adapt to patient needs and ensure equitable access. Future research should examine the impact of relational familiarity and the role of systemic reforms to enhance palliative care.

## Background

The global demographic shift toward an ageing population, combined with increased life expectancy and multimorbidity, has led to a growing need for palliative care in primary healthcare settings.^1,2^ Alongside this trend, there has been a rising emphasis on delivering healthcare services in patients’ homes, reflecting the preference of many individuals to spend their final days in familiar and comforting surroundings.^3,4^ In Norway, this shift towards home-based care is increasingly evident. Policymakers and healthcare stakeholders advocate that patients remain at home longer, even during the final stages of life.^5,6^ Consequently, the demand for competent, coordinated palliative care services in the municipalities is expected to rise significantly in the coming decades.^7,8^

One such service is the general practitioner (GP) system, which ensures that every resident has access to a regular, long-term GP.^9^ This system serves as a cornerstone of palliative care for home-dwelling patients in Norway. Long-term relationships with GPs are associated with better outcomes, including reduced mortality and fewer hospital admissions.^10,11^ Continuity and stability in the doctor–patient relationship are fundamental for delivering high-quality care, particularly for older adults whose reliance on GP services intensifies with age.^12,13^ Conversely, a disruption in continuity, such as changes in GPs or poor communication between healthcare professionals, can exacerbate older adults’ vulnerability and lead to avoidable hospitalisation.^8^

In Norway, GPs are tasked with a wide range of responsibilities, including acute and chronic care, preventive medicine and palliative care, which contribute to an already heavy workload. A long-standing GP shortage further strains access to primary care.^14^ At the same time, organisation changes, such as group practices, part-time arrangements and reliance on locums, further challenge continuity and coordination.^15–17^ In addition, local variations within Norway in how palliative care is organised and practiced, for example, differences in the availability of cancer care coordinators and specialised palliative home-care teams, 24/7 home nursing coverage and GP list sizes, contribute to gaps between guidelines and everyday practice.^18^ Moreover, GPs often report feeling disconnected from broader medical follow-ups and face challenges such as time-consuming home visits, limited specialised competence and infrequent contact with older home-dwelling patients with palliative care needs.^19,20^ However, while these demands can be a source of psychological stress,^21^ many GPs find home-based palliative care meaningful and fulfilling, reflecting a sense of social responsibility and a commitment to the population.^22^

Existing research has explored the roles and expectations of GPs in primary healthcare and palliative care,^23–26^ highlighting the need for further investigation into organisational barriers, the evident challenges of care continuity and interprofessional collaboration. Studies on GPs’ collaboration with specialised palliative home care emphasise the importance of physicians’ availability and sufficient time for patient and family care.^27,28^ From a policy perspective, GPs are among the core figures in person-centred care.^29,30^ GPs are often viewed as coordinators and key figures in home-based palliative care, possessing unique relational knowledge of patients and families that other healthcare professionals often lack.^31^ Research also shows that relational continuity fosters trust and security for patients and families and is associated with better outcomes, including care aligned with patient preferences,^32^ and may also influence decision-making processes.^33^

Hence, the extent of GP involvement in palliative care is often influenced by contextual factors, such as time availability, organisational structures and geography. For example, home visits by GPs are more likely to occur in rural areas.^34,35^ While much research has focused on the roles and systemic challenges faced by GPs in palliative care, studies of the relational aspects have primarily examined how familiarity and continuity build trust and improve care quality.^36,37^ Far less attention has been given to whether this same familiarity might also introduce a bias – where assumptions based on standing relationships affect clinical judgement and individualised care. With GPs’ central role in person-centred care, understanding how they navigate the interpersonal dimension of their work is crucial because these relationships are at the core of delivering palliative care. Thus, this article aims to explore how GPs navigate the relational dimensions of their work when caring for home-dwelling patients in the palliative phase, and whether relational familiarity serves as a basis – or a bias – for individualised care.

## Methods

### Design

This study utilised an exploratory, descriptive and qualitative research approach grounded in hermeneutic methodology.^38^ The research involved a secondary analysis of data originating from two previously completed studies. These datasets were selected because both elicited GPs’ accounts of relational familiarity in home-based palliative care, providing complementary perspectives directly aligned with our question of whether familiarity functions as a basis of bias for individualised care.^39,40^ The two primary studies were independently conducted by the first and second authors in 2020 and 2024, respectively, and will be referred to as the primary studies in this article. The secondary analysis incorporated data from 21 GPs who participated across both studies (see Table 1). Because the objective of secondary analysis is to explore new research questions using pre-existing data, it is recommended to apply analytical techniques that align closely with those utilised in the original research. In this study, we adhered to Gadamer’s philosophical hermeneutics to guide interpretation because it treats understanding as a dialogical event between interpreter and text, shaped by historically effected consciousness. Rather than bracketing prior assumptions, Gadamer invites their explicit articulation and testing against the subject matter through the hermeneutic circle, an iterative movement between parts and whole, culminating in a fusion of horizons.^38,41^ This process allowed us to develop a deeper and more nuanced understanding of textual material while refining and adjusting our preunderstanding throughout the interpretational process.

**Table 1.** Study participants’ socio-demographic data.

	Characteristics of study participants	
	Dataset 1 (*N* = 7)	Dataset 2 (*N* = 14)
Female	3	6
Male	4	8
Age	4	8
<40 years	1	6
41–50 years	3	3
51–60 years	3	4
61–70 years	0	1
71–80 years	0	0
Years of work experience		
<5 years	0	2
5–10 years	4	2
11–15 years	1	6
16–20 years	1	1
>20 years	1	3

### Participants and data collection of the primary studies

In both primary studies, GPs were interviewed regarding their experiences with providing care to older adults living at home in palliative care contexts. The data collection focused on, respectively, their experiences with dignity-preserving care and decision-making processes for this patient group. The primary studies included cancer coordinators and GPs as informants, investigating both shared experiences and unique perspectives separately. During the interpretation phase of the primary studies, it became apparent that a substantial portion of the data gathered from GPs had not been fully explored. Correspondingly, the present study exclusively targets transcribed raw data from GPs. These data offer rich insights into the dynamics of the relationship between GPs and older home-dwelling patients, providing essential knowledge about how GPs navigate the relational dimensions of their work when caring for home-dwelling patients in the palliative phase. Therefore, we combined the transcripts of these two hermeneutic qualitative studies that addressed complementary perspectives of person-centred palliative care in Norwegian primary care. Both used semi-structured guides grounded in Gadamerian hermeneutics and elicited reflections on enduring relational and organisational features of home-based palliative care. Using only GP data ensured a shared professional lens and given the richness of the material from 21 GPs, we judged the combined sample sufficient for the aim of this secondary hermeneutic analysis. Also, the difference in interview format provided complementary depth and breadth.

To clarify the context and methodological framework of the original data collection, a brief overview of the two primary studies is presented below.

Dataset 1: *Healthcare professionals’ perceptions of dignity-preserving care for older home-dwelling women with incurable cancer in Norway*.^19^

Grounded in Gadamer’s philosophical hermeneutics,^38^ this work sought to investigate healthcare professionals’ (cancer coordinators and GPs’) perceptions of conditions contributing to dignity-preserving care for older home-dwelling women with incurable cancer. Data collection included three focus group interviews with cancer coordinators and seven in-depth interviews with GPs conducted between March and June 2020. For this study, only the in-depth interviews with GPs were emphasised. Inclusion criteria were being a GP and having experience with the medical care of older home-dwelling women with incurable cancer. GP recruitment employed a strategic sampling approach supplemented by the snowball method to identify GPs responsible for such care.^42^ Participants were drawn from four municipalities representing both urban and rural settings. Due to COVID-19 restrictions, interviews in 2020 were conducted via digital platforms and telephone. We consider this a procedural rather than substantive influence, as the interview guide did not include pandemic-specific questions and participants were encouraged to reflect on their long-standing practice experiences. A semi-structured, modifiable interview guide allowed flexibility during the interviews, with questions such as: ‘What do you experience as fundamental to safeguarding the dignity of older home-dwelling adults who wish to spend their final days at home?’ All seven interviews, lasting between 43 and 60 min, were recorded and transcribed verbatim.

Dataset 2: *Navigating together towards shared decision making in home-based palliative care – perspectives from general practitioners and cancer care coordinators* (Svendsen SJ, Grov EK, Staats K: manuscript under review at BMC Health Services Research).

This study used a hermeneutic methodology^38^ examining the experiences of GPs and cancer coordinators with shared decision making in palliative care for home-dwelling patients. The interpretation of the interviews followed the approach outlined by Fleming et al.^43^ Data collection included two group interviews with cancer coordinators and two focus group interviews with GPs. Only focus groups involving GPs were assessed. Inclusion criteria were being a GP and having experience with the medical care of home-dwelling patients in the palliative phase. Participants were recruited through a snowball sampling method facilitated by a national research network of palliative caregivers in primary healthcare. The two focus groups included eight and six GPs, respectively, and represented both urban and rural municipalities. Interviews were conducted at the participants’ workplace using a semi-structured, modifiable interview guide. A notable feature of these interviews was the presentation of a quote from a homecare nurse drawn from a prior study^44^ that described an undignified care situation. This quote stimulated discussion and prompted deep reflection among the GPs. Both focus group interviews were recorded and transcribed verbatim, with durations ranging from 65 to 80 min.

### Second analysis using the hermeneutical circle

To achieve a comprehensive understanding of the data, all authors engaged in a circular hermeneutical process that involved thoroughly and repeatedly reading the clean, uncoded transcripts.^38,45^ This iterative method emphasises continuous dialogue between the whole and its parts, where each element informs and enriches the interpretation of the other.^46^ We began by independently reading and coding the transcripts, sharing reflections and documenting preliminary observations in a common file. Each dataset was analysed separately before comparison, with excerpts tagged by dataset and method. We then repeatedly returned to our individual analyses to reassess codes in light of emerging insights, before jointly determining the thematic structure through consensus. This collaborative cycling between solitary and collective work enabled critical scrutiny of interpretations and fostered a deeper, more robust understanding of the data. Particular attention was given to identifying both overlapping and contradictory interpretations across the two datasets. We judged the datasets comparable because they involve the same professional group and care context. Differences in focus and format were addressed through separate interpretation before comparison, which enriched rather than undermined the secondary analysis. Subsequently, we revisited the datasets, now informed by a more nuanced understanding of their individual parts, and began identifying preliminary emerging patterns of meaning. During this stage, we remained mindful of our pre-understandings as we explored and formulated themes and subthemes, ultimately arriving at what we considered a final interpretive understanding of the data.

### Pre-understanding

As researchers, our preunderstanding was neither neutral nor detached but shaped by a committed relationship to the subject matter. All authors are registered nurses with diverse professional and research experience in palliative care and existential issues. To ensure transparency and enhance the trustworthiness of our study, we actively discussed and critically examined our pre-understanding throughout the research process.^47^ Our research team comprised an associate professor with formal and clinical expertise in palliative care, a PhD candidate with substantial clinical experience in the same field and a mental health nurse with both clinical and research expertise in addressing existential and relational concerns for patients in vulnerable life circumstances. In addition, all authors brought extensive experience in collaborative work with GPs, including a knowledge of their diverse roles in palliative care and the challenges they face in balancing the needs of patients with varied diagnoses. Our preunderstanding was initially grounded in the assumption that GPs often operate on the periphery of care for home-dwelling patients in a palliative phase. We also presumed a significant degree of undefined responsibility in the collaborative processes surrounding these patients. However, as we progressed through the hermeneutical interpretation phase, the participants’ insights challenged these preconceptions. This process led to a revised understanding and allowed us to uncover deeper meanings of the subject matter.

### Ethical considerations

The reuse of data is particularly significant when the target group is challenging to access in primary care settings.^48^ This approach helps minimise the burden on informants and reduces the frequency with which they are asked to participate in research.^40^ Thus, we deemed it ethically responsible for merging the GP data from two primary studies. When entrusted with valuable knowledge shared by others, we believe that there is an ethical obligation to disseminate this knowledge to the broader community. In the primary studies, all participants were provided with both written and oral information about the research and gave written consent for their participation. Respondents were also informed of their right to withdraw from the study at any time without providing an explanation. The studies were conducted in full compliance with the ethical principles outlined in the Declaration of Helsinki^49^ and the Norwegian Health Research Act.^50^ The Norwegian Agency for Shared Services in Education and Research provided ethical approval of the primary studies (ref. no. 138698 and 732136).

## Results

The interpretation of the data from the participants revealed a complex and nuanced picture of the relational perspective of palliative care between GPs and home-dwelling patients. Across the material, two overarching themes emerged: *relational familiarity: A cornerstone with complex dimensions* and *variability in engagement and follow-up.* Each will be discussed in turn below.

### Relational familiarity: A cornerstone with complex dimensions

Relational familiarity emerged as a cornerstone of effective palliative care, deeply influencing how the participants approached their patients’ final phases of life. When the informants were familiar with the patients and their families, it enabled them to anticipate needs, tailor communication and support decision making better. Familiarity was perceived as particularly crucial to facilitating home death, where trust and continuity were considered essential. According to most GPs, familiarity was not just a matter of knowing the patient – it was about being present and willing to engage with the ethical complexities of palliative care. The findings illustrate many dimensions of familiarity and how this manifested differently across care trajectories. For example, it allowed the participants to rely on professional judgement rather than rigid guidelines, especially when those guidelines conflicted with what they knew to be important to the patient. As one participant shared:

The patient was nearing the end of life, and while it was agreed the son would stay with him, I realized he wasn’t coming. I knew the patient well – he wasn’t afraid to be alone, was pain-free and unlikely to face a crisis. If I told the healthcare team, they would insist on moving him to a nursing home, which he didn’t want. So, I chose to stay quiet. (GP1, Dataset 1)

However, familiarity has two sides. As described, the GPs acknowledged that familiarity fosters meaningful interactions between the GP and the patient, enabling good follow-up care. As such, several participants emphasised that this could reduce the need for frequent home visits, ease the collaboration with the next of kin and prevent unnecessary hospital admissions. Conversely, familiarity can feel similar to a burden. Some participants expressed concern about becoming too emotionally close, fearing they might overstep boundaries or struggle with the ethical weight of end-of-life decisions. One respondent described how patients and their families who feel deeply connected to them may come to rely entirely on them, which can limit their ability to take a step back or delegate tasks:

You end up involved with the whole family. I was the GP for her mother and her husband. I also had her son. So, I knew the whole family. And that affects the entire trajectory, doesn’t it? The mother had anxiety attacks and was with her daughter when she was at home in pain after the surgery. So, it has its ripple effects, and the husband had some back issues. . . . It was also something psychological. He was exhausted and . . . the whole situation, really. (GP5, Dataset 1)

Another dimension of the interviewee’s perspective of familiarity was how GPs experienced the expectations and ideals tied to their roles, both from patients and other healthcare professionals. The GPs described how a lack of familiarity with the patient could make it harder to be fully present and attentive to the patient’s actual needs and situation. In such cases, they were likelier to be guided by their own and others’ expectations of their role, rather than adapting to the patient’s unique process. This could create challenges in balancing acting and waiting, potentially reducing their sensitivity to the patient’s individual needs. As one participant shared:

I took it upon myself to act as a “reality adjuster” and visited a patient at home, even on my day off, to address her unrealistic expectations. During our conversation, I gently questioned her assumptions, which led to a productive discussion at the time. However, afterward, stories circulated that I had taken away her hope, and both she and her family no longer wanted to see me. (GP2, Dataset 1)

This delicate interplay between familiarity, expectations and professional boundaries highlights the complexity of the GP’s role. Building on this, in one of the focus groups, the GPs discussed that it is acceptable not to always have complete familiarity with the patient, especially when others are involved in their follow-up care. Patients sometimes become overly dependent on the GP, which is why some GPs choose to take a more cautious and reserved approach. This could be inviting a colleague’s input and briefly stepping back so that new perspectives could occur, underscoring that this overfamiliarity could be a potential bias for individualised care.

They agreed that familiarity can also mean trusting that other healthcare professionals have the necessary knowledge and familiarity. While this trust was generally seen as positive, enabling delegation and shared responsibility, as the following quote illustrates, it could occasionally blur the lines of responsibility, making it less clear who should take the lead. which sometimes leads to unclear lines of responsibility:

The homecare nurses often know the patient well – their functional level, their illness and their level of frailty – through daily contact. I think, in general, it’s important that those who know the patient well – who understand how frail the patient is or isn’t and perhaps know what the patient themselves has said – should play a bigger role’. . . . ‘Yes, any work you can delegate to others is very helpful. Then you also have someone to support you, someone who knows the patient and has been involved. In that way, I’m not the first one who must bring things up’. (Focus Group 2, Dataset 2)

In this sense, trusting others’ familiarity, whether from nurses or GP colleagues, was described as bringing ‘fresh eyes’ that could recalibrate plans when overfamiliarity risked blunting sensitivity to change. While familiarity and trust were seen as important, the material also revealed considerable variation in how GPs engaged with palliative care and followed up with patients.

### Variability in engagement and follow-up

The participants described a wide range of practices for engaging with palliative care and following up with home-dwelling patients. This variability was shaped by individual interests, systemic constraints and the presence of other actors in the care network. As some participants mentioned, a key factor influencing engagement was their ability to collaborate and stay proactive in the care trajectory, applying scenario thinking some respondents emphasised the importance of establishing a so-called “fire escape plan” – a flexible Plan B that could be implemented if the home situation changed or became unsafe. However, most informants reported being excluded from opportunities to build familiarity and co-create care plans, especially when specialist services became involved. Although this exclusion was often accepted as practical, it still represented a loss of involvement for many participants. In these cases, they often relinquished their role, sometimes reluctantly when they felt sidelined, and at other times as a pragmatic choice to manage due to the complexity and time demands of palliative care. From one focus group:

We often feel less involved, but trust that the patients are well cared for by the cancer care coordinator. When they are involved, I feel confident in their thorough follow-up and that I will be contacted if needed. Ideally, I’d like to be more involved, but with many patients and limited time, prioritization is key. When we have other actors who are specialists in this, I think it’s fine for us to stay a bit on the sidelines in these processes. (Focus Group 1, Dataset 2)

The variability in engagement and follow-up was closely tied to whether the participants were genuinely interested in palliative care. Those with a strong interest tended to provide close and continuous support, while others felt more relieved when stepping back, trusting that specialised healthcare professionals would ensure that the patient received adequate help. The idea of a ‘recipe for success’ was frequently mentioned; however, it was perceived as highly personal and dependent on the GP’s individual interests and capacity. Hence, the participants acknowledged that this could lead to significant variations in the quality and intensity of follow-up, particularly regarding the possibility of dying at home. As one participant explained:

It becomes somewhat random, doesn’t it? Some patients receive more follow-up from me, and others receive more follow-up from the hospital. But I find that if they don’t feel they have that connection with the hospital department, they turn to me. Or if they already have a very good relationship with me, they come to me. And if not, they feel well cared for by the hospital to a sufficient degree. So, we can complement each other. (GP7, Dataset 1)

Several GPs explicitly linked this variability to practice configurations, such as part-time contracts, group-practice rosters and locum coverage. This constrained availability for home visits and continuity, and at times shifted the GP from coordinator to occasional contributor. At the same time, group and part‑time arrangements occasionally enabled complementary perspectives, another GP in the group or a locum noticing changes that the regular, closely familiar GP might overlook. However, some interviewees described themselves as just a link in the system rather than central figures in the care trajectory. They noted that decisions made in hospitals were often left untouched even when they had relevant knowledge or insight:

So, in a way, we end up standing a bit on the sidelines. We become something of a secretary, in a sense. Others, such as the hospital, come in, make the decisions and set the plans, and then we are asked, for example, to write some prescriptions. That is perhaps the most common scenario. (Focus Group 1, Dataset 2)

Other participants reflected on whether they needed to know everything, such as the contents of medication kits or the details of symptom management at the end of life – or whether nurses with specialised competence could play a more prominent role. While familiarity was usually a basis for individualised care, in some cases it risked bias though overfamiliarity. Despite this, some participants emphasised that good collaboration could compensate for limited direct involvement. For them, stepping back did not necessarily mean neglecting the patient – as long as communication was clear and responsibility was shared. Several participants emphasised that alternating or shared GP follow-up in smaller groups could ‘shed new light’ on patient needs and help mitigate blind spots arising from overfamiliarity.

## Discussion

The results illuminated the complex relational aspects that GPs encounter when providing palliative care to home-dwelling patients. Relational familiarity was identified as both a strength and a challenge, fostering trust, continuity and personalised care while raising concerns about boundaries and role expectations. Meanwhile, variable engagement reflected differences in GPs’ individual interests, systemic constraints and the involvement of other healthcare professionals. Together, these themes underscore the dynamic interplay between personal commitment, professional boundaries and collaborative care in shaping the quality and consistency of palliative care delivery. We will discuss this in relation to previous research and the principles of person-centred care, targeting relational familiarity as a *basis* and a *bias* for individualised care.

### Relational familiarity as a basis for person-centred care

Relational familiarity emerged in this study as a cornerstone of palliative care. When GPs knew their patients and families well, they could anticipate needs, tailor communication and support decision making in ways that aligned with the patient’s needs, values and preferences. This relational depth was particularly crucial for facilitating home deaths, where trust and continuity were believed essential. Participants described how familiarity enabled them to rely on professional judgement rather than strictly adhering to rigid guidelines, especially when those guidelines conflicted with what they knew was important to the patient – a finding that resonates with person-centred care principles.^29^ Person-centred care resists standardisations and cannot be reduced to a fixed set of predefined functions or responsibilities.^51^ Each palliative trajectory is inherently unique and shaped by the distinct individuals and circumstances involved. As such, what constitutes person-centred care in one context may diverge from what is needed in another.^52^ Relational familiarity enabled GPs to act with greater confidence and flexibility, often reducing the need for frequent home visits, easing collaboration with other healthcare professionals and family members and preventing unnecessary hospital admissions. Several participants emphasised that being emotionally attuned and present was not only beneficial but also ethically essential when navigating the complexities of palliative care. This perspective aligns with research showing that palliative care is inherently relational and benefits from a broad person-centred approach grounded in trust built over time and attention to what matters to patients and families.^23,30^ Notably, familiarity was not a static quality; the participants in our study emphasised the need for flexibility, which manifested in different ways across care trajectories. Some GPs described using scenario thinking to develop “fire escape plans” – flexible strategies for responding to changes in the home situation – and how familiarity helped them co-create care plans with patients and families. In these cases, relational continuity empowered GPs to act in alignment with what mattered most to the patient, even when this meant deviating from established protocols. This reflects on what Kitson et al.^29^ describe as the therapeutic relationship at the heart of person-centred care, where clinical decisions are shaped by a deep understanding of the patient’s context. Patients who benefit from long-term continuity with their GPs – often termed ‘family doctors’ – tend to receive more personalised and coordinated care,^32^ further reinforcing the value of relational familiarity as the basis for person-centred care.

### Relational familiarity as a bias for person-centred care

While relational familiarity often serves as a *basis* for high-quality, person-centred care, this study also highlights how its potential can act as a *bias*, contributing to inequities in healthcare delivery. As this study found, several participants disclosed that becoming too emotionally and professionally close to patients and their families could feel burdensome. This closeness made it difficult to maintain professional boundaries – such as keeping a clear distinction between personal involvement and clinical responsibility – and to delegate tasks to other healthcare professionals. Paradoxically, the resulting emotional strain sometimes led GPs to withdraw, creating a distance and a loss of familiarity. This aligns with research showing that patients without a long-standing personalised relationship with their GP are more likely to experience fragmented, impersonal and less person-centred care, exacerbating disparities in access and outcomes.^53^ Conversely, our study indicates that overfamiliarity can bias individualised care and illustrate how inviting “fresh eyes” from others can counter potential blind spots and strengthen trust. These dynamics underscore a critical tension: while relational familiarity is essential for person-centred care,^29^ it can also inadvertently reinforce inequities. In our material, inequities were amplified by practice arrangements, part-time schedules, group-practice rotas and reliance on locums, which constrained availability, continuity and proactive follow-up.

In the Norwegian context, the GP scheme^9^ is designed to promote continuity of care and support relational familiarity. Nonetheless, relational familiarity is unevenly distributed and influenced by factors such as geographic location, practice capacity and patient mobility.^54^ For instance, rural areas may foster closer GP–patient relationships due to smaller patient populations and greater continuity, while urban areas with higher patient turnover may limit the opportunity to cultivate such familiarity.^54^ These structural inconsistencies in healthcare systems can foster perceptions of social injustice, particularly in palliative care, where relational familiarity often becomes the cornerstone of person-centred planning and decision making. In home-based palliative care, continuity is often disrupted by the system and context.^53,55^ Kitson et al.^29^ note that, while there is broad agreement on what person-centred care means (seeing the whole person, partnering in decisions, attending to context), different professions apply it differently in practice. This leads to variation in delivery and can undermine equity. A strong GP–patient relationship is therefore important, but on its own, cannot overcome organisational barriers; equitable person-centred care also requires coordinated teams and supportive systems.^29^

Although international^56,57^ and national^58^ guidelines underscore the importance of person-centredness in palliative care, they often fail to account for the practical realities faced by GPs. Research indicates that GPs frequently encounter barriers such as discontinuity in GP–patient relationships, insufficient time and limited resources to maintain relational familiarity, often linked to part-time work patterns or group-practice arrangements.^24^ These constraints can make it difficult for GPs to provide equitable care to all patients, particularly those with complex needs who require more individualised attention. Our findings link these organisational realities to how familiarity is distributed and to when overfamiliarity may arise, thereby connecting micro-level relational dynamics with macro-level constraints.

Ultimately, this raises the question of whether the GP must necessarily be the key figure in providing a person-centred palliative care for home-dwelling patients. While the GP traditionally holds a central role in primary healthcare, person-centred care challenges established hierarchies by asking not *who* holds the formal responsibility, but *who* is best positioned to understand and act in alignment with the patient’s values, needs and priorities. If the essence of person-centred care lies in relational familiarity and responsiveness, then the role should fall to the professional who is closest to the patient – regardless of their formal authority or predefined roles within the healthcare system. Interdisciplinary collaboration, when grounded in shared understanding and mutual trust, has proven effective at delivering holistic, tailored support.^59,60^ The findings from this study show that GPs, themselves, expressed ambivalence about their role in palliative care. Some questioned whether they were indeed the most appropriate person to take the lead in person-centred care, given the structural limitations of their everyday practice.

This tension suggests that person-centred care may require a redefinition of roles – where proximity, trust and relational familiarity are prioritised over professional boundaries. Rather than assuming the GP must always be the central figure, care models should allow dynamic role distribution based on who can best meet the patient’s needs in a given context. Practically, this entails deliberate use of ‘fresh eyes’ (alternating/shared GP follow-up in small groups), explicit scenario thinking, and clearer delegation to the professional with the closest, most relevant familiarity at a given time.

## Methodological considerations

We found secondary analysis a suitable method for combining data from the two primary studies in this article. This approach allowed us to revisit and analyse unexplored aspects of rich qualitative data, avoiding the need to engage new participants while maximising the value of existing material.^61^ Secondary analysis is particularly valuable when studying sensitive topics or hard-to-reach populations, as it minimises participants’ burdens and fosters cumulative rather than repetitive research processes.^40,62^ In this study, the datasets centred on GPs’ experiences with dignity-preserving care and shared decision making in palliative contexts, offering complementary insights into the relational dimensions of care. Involving multiple researchers in the interpretive process was crucial, as meaning emerged from the collaborative analysis rather than residing inherently within the data.^63^ However, some methodological limitations must be acknowledged. We did not conduct formal pilot interviews in either dataset; instead, the interview and focus group questions were tested for clarity with academic colleagues with relevant healthcare backgrounds. Moreover, secondary analysis may lack the contextual familiarity of primary research, as new researchers are not immersed in the original data collection process. Data interpretation can evolve over time due to shifts in researchers’ perspectives.^64^ While efforts were made to remain reflexive and transparent, we cannot fully account for how participants’ responses might have varied with other researchers or under different circumstances. In addition, contextual variations between the two primary studies, such as distinctions in participant demographics and interview settings, may have influenced the data. Furthermore, the primary studies targeted different patient groups (older women with incurable cancer vs a broader home-dwelling palliative population), which may have shaped GPs’ examples and emphases; this heterogeneity might be a limitation, and we therefore interpret dataset-specific nuances cautiously. Additionally, the 2020 interviews were conducted digitally/by telephone due to COVID 19 restrictions; this affected interview modality only, and neither the research questions nor the secondary hermeneutical interpretations were influenced. Despite these challenges, secondary analysis provided valuable opportunities to generate new insights into the relational dynamics of GP palliative care practices.

## Conclusion and implications

Relational familiarity is usually a basis for individualised, person-centred palliative care, building trust, continuity and decisions aligned with what matters to patients. Yet overfamiliarity can become a bias, narrowing perspective, blurring boundaries and contributing to inequities, especially under practice arrangements such as part-time schedules, group-practice rotas and reliance on locums. To retain benefits and mitigate risks, care should deliberately incorporate fresh eyes (alternating/shared GP follow-up in small groups), explicit scenario thinking and clear delegation to the professional with the closest, most relevant familiarity at a given time, supported by coordinated interdisciplinary teams and systems. Policymakers should address organisational barriers to continuity and equitable access and enable flexible models that distribute familiarity more fairly. Future research should explore the role of relational familiarity in care outcomes, the dynamics of interdisciplinary collaboration and how systemic reforms can optimise person-centred palliative care for home-dwelling patients.

## Supplemental Material

sj-docx-1-pcr-10.1177_26323524261426398 – Supplemental material for General practitioners’ relational familiarity with home-dwelling patients in the palliative phase: Basis – or bias – for individualised care Supplemental material, sj-docx-1-pcr-10.1177_26323524261426398 for General practitioners’ relational familiarity with home-dwelling patients in the palliative phase: Basis – or bias – for individualised care by Katrine Staats, Sandra Jahr Svendsen and Veronica Lockertsen in Palliative Care and Social Practice
